# Cell plasticity in cancer and therapy resistance

**DOI:** 10.1038/s12276-026-01869-y

**Published:** 2026-10-07

**Authors:** Yoojeong Seo, Jinho Jang, Jae-Il Park

**Affiliations:** 1 Department of Experimental Radiation Oncology, Division of Radiation Oncology, The University of Texas MD Anderson Cancer Center, Houston, TX USA; 2 Graduate School of Biomedical Sciences, The University of Texas MD Anderson Cancer Center, Houston, TX USA; 3 Program in Genetics and Epigenetics, The University of Texas MD Anderson Cancer Center, Houston, TX USA

**Keywords:** Tumour heterogeneity, Cancer therapeutic resistance

## Abstract

Cancer cell plasticity, the ability of tumor cells to switch between distinct cellular states, has emerged as a key mechanism driving tumor progression, metastasis and therapy resistance. Cancer cells exploit diverse plasticity mechanisms, including dedifferentiation, transdifferentiation, epithelial–mesenchymal transition and stem cell-like reprogramming, to adapt to changes in their microenvironment or therapeutic pressures. These transitions are orchestrated by signaling pathways, epigenetic modifications and microenvironmental cues. Notably, plasticity encompasses reversible transitions among intermediate states that enhance metastatic potential and contribute to therapeutic failure. Tumor cells can enter drug-tolerant reversible states, enabling survival during treatment and leading to relapse. Recent advances in single-cell and spatial transcriptomics, along with conceptual frameworks such as the Waddington landscape, have provided new insights into the dynamic regulation of tumor cell-state transitions at high resolution. In this Review, we discuss the molecular and cellular mechanisms governing cancer cell plasticity, its impact on metastasis and therapy resistance, and recent technological advances that illuminate dynamic tumor cell-state transitions.

## Cancer cell plasticity drives metastasis and therapy resistance

Cancer cell plasticity, the ability of tumor cells to transition between various phenotypic states, plays a crucial role in cancer progression, metastasis and therapy resistance. This Review explores how these shifts among differentiated, stem-like, proliferative, invasive, dormant and drug-tolerant states contribute to intratumoral heterogeneity. The authors discuss the genetic, epigenetic and microenvironmental factors driving these phenotype switching. Key findings include the identification of hybrid cell plastic states that enhance metastatic potential and therapeutic evasion. Furthermore, the Review highlights the importance  of single-cell and spatial transcriptomics in decoding these dynamic processes, offering insights into potential therapeutic strategies targeting plasticity. Future directions involve exploiting cancer cell plasticity to prevent metastasis and overcome therapy resistance, aiming for more durable clinical responses.

This summary was initially drafted using artificial intelligence, then revised and fact-checked by the author.

## Introduction

Cell plasticity refers to the capacity of a cell to alter its phenotypic and functional identity in response to developmental, homeostatic, injury-associated or environmental cues. In adult tissues, this flexibility involves changes in differentiation state, including cell dedifferentiation, transdifferentiation or the acquisition of progenitor-like features, and contributes to tissue homeostasis and regeneration. Although cellular identity is constrained by lineage-specific regulatory networks, it is not invariably fixed, and contextual signals can redirect cell fate under physiological and pathological conditions^1–3^.

In cancer, these normally adaptive programs are co-opted to support tumor initiation, progression, metastasis and resistance to therapy^4–6^. Cancer progression has traditionally been understood through genetic and clonal evolution, which emphasize the selection of genetically distinct clones carrying fitness-enhancing alterations^7,8^. Cancer cell plasticity extends rather than replaces these concepts by highlighting the ability of tumor cells to undergo reversible, nongenetic transitions between phenotypic states^4,5^. Although genetic alterations may establish a permissive background for tumor evolution, they do not fully explain the phenotypic heterogeneity and rapid adaptability of cancer cells under environmental and therapeutic pressures^4,5,8,9^.

Recent advances in cell lineage-tracing, single-cell and spatial transcriptomics have enabled dynamic cell states, lineage trajectories and their microenvironmental contexts to be examined at unprecedented resolution^10–13^. Waddington’s classical landscape provides a complementary conceptual framework in which cellular phenotypes are viewed as stable or semi-stable states that cells tend to maintain, whereas plasticity represents movement between these states^14–16^.

In this Review, we discuss the cellular and molecular mechanisms governing cancer cell plasticity, its roles in tumor initiation, metastasis and therapeutic resistance, and emerging technologies and therapeutic strategies for studying and targeting adaptive cell-state transitions.

### Cancer cell plasticity

Cancer cell plasticity is the ability of tumor cells to dynamically and often reversibly transition between differentiated, stem-like, proliferative, invasive, dormant and drug-tolerant states^4–6,17,18^. Rather than being confined to a rare or predefined subset of tumor cells, stem cell-like and more differentiated cancer cells can interconvert in response to contextual signals, thereby generating dynamic intratumoral heterogeneity^6,17,19^.

These state transitions support metastatic dissemination and colonization by allowing tumor cells to alternate between invasive and proliferative phenotypes^20–23^. They also promote therapy resistance and relapse by enabling transient drug-tolerant or alternative lineage states^6,9,18,23^.

### Drivers of cancer cell plasticity

**Fig. 1:**
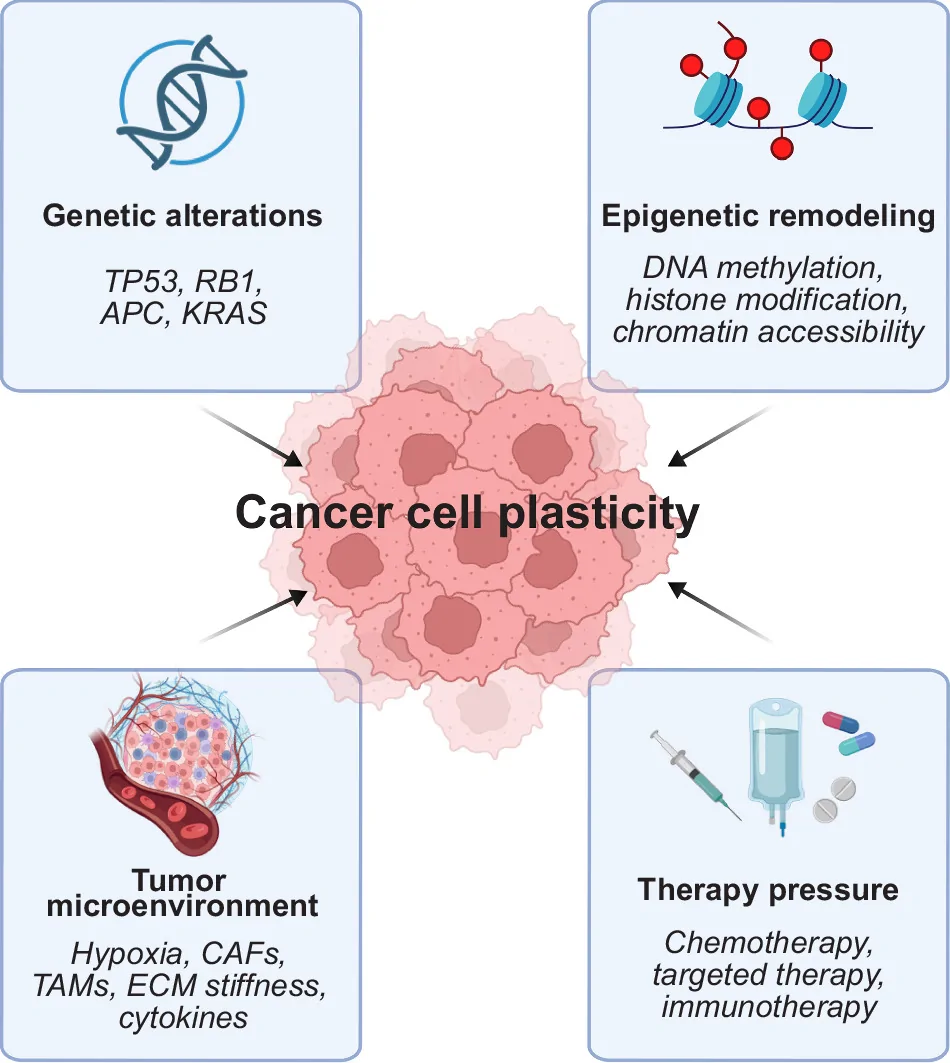
Drivers of cancer cell plasticity. Cancer cell plasticity is regulated by multiple interconnected intrinsic and extrinsic factors. Genetic alterations, including mutations in *TP53*,* RB1*,* APC* and *KRAS*, can establish a permissive background for cancer cell plasticity. Epigenetic remodeling through DNA methylation, histone modifications and changes in chromatin accessibility enables dynamic and reversible transitions between cellular states^18^. The TME, including hypoxia, CAFs, TAMs, ECM stiffness and inflammatory cytokines, further shapes plastic phenotypes through biochemical and mechanical signaling. In addition, therapeutic pressure imposed by chemotherapy, targeted therapy and immunotherapy promotes adaptive cell-state transitions that contribute to drug tolerance and resistance. Together, these intrinsic and extrinsic factors drive dynamic cell-state transitions associated with tumor progression, metastasis and therapy resistance.

Cancer cell plasticity is shaped by interconnected genetic, epigenetic and microenvironmental factors^23,24^ (Fig. 1). Genetic alterations can establish a permissive background for plasticity by disrupting lineage constraints, as exemplified by mutations in key tumor suppressors or developmental regulators that facilitate cell state transitions^23^. However, genetic events alone are often insufficient to fully explain dynamic and reversible phenotypic changes^5^.

Epigenetic regulation plays a central role in promoting plasticity by controlling chromatin accessibility and transcriptional programs without altering the underlying DNA sequence^24^. Changes in histone modifications, DNA methylation and chromatin remodeling can facilitate switching between cellular states, allowing tumor cells to adapt to hostile stress and therapeutic pressure^18,24^. These mechanisms are particularly important for establishing drug-tolerant states and promoting cell lineage reprogramming^18^.

In addition, signals from the tumor microenvironment (TME) provide critical external inputs that shape cell-state dynamics^5,25^. Interactions with stromal cells, immune components, and the extracellular matrix (ECM) activate signaling pathways that promote invasion, survival and immune evasion, thereby reinforcing plastic phenotypes^5,25^. Tissue injury and regeneration programs, including those associated with embryonic development, can be aberrantly activated in tumor cells, driving transitions toward progenitor-like or stem cell-like states^1,4,5^. Such processes have been implicated in early tumorigenesis, including preneoplastic stages, where increased cellular plasticity may precede full malignant transformation^1,26,27^.

### Cancer-specific manifestations of cell plasticity

Cancer cell plasticity has been extensively studied in breast cancer, where epithelial–mesenchymal transition (EMT) and cancer stem cell (CSC) programs emerged as early and influential models linking plasticity to metastasis and therapeutic resistance. Studies from the Weinberg group^28,29^ and others demonstrated that induction of EMT in mammary epithelial cells generated stem cell-like properties, enhanced invasiveness and increased tumor-initiating capacity^20,29–31^. These findings expanded the role of EMT beyond migration, linking it to cellular dedifferentiation and metastatic competence. Subsequent studies further showed that hybrid epithelial/mesenchymal states can retain both migratory and proliferative capacities, suggesting that partial EMT states may be particularly important for metastatic dissemination and therapy resistance.

In lung adenocarcinoma, recent studies have highlighted the existence of highly plastic cellular states associated with therapeutic adaptation and resistance^32,33^. Beyond classical histologic transformation, single-cell analyses have revealed transitional populations capable of dynamically shifting between epithelial, mesenchymal, regenerative and drug-tolerant phenotypes under treatment pressure. These therapy-adaptive states are associated with extensive transcriptional and epigenetic remodeling and can persist despite targeted therapy exposure, supporting the concept that resistance may emerge through reversible cell-state transitions rather than solely through genetic selection.

Neuroendocrine (NE) plasticity in lung cancer represents a particularly striking example of lineage switching^34^. In EGFR-mutant lung adenocarcinoma, targeted therapy or actin dysregulation can promote transformation into small-cell lung cancer (SCLC)-like states characterized by NE marker expression, loss of lineage fidelity, and aggressive clinical behavior^34,35^. NE plasticity contributes to the aggressive behavior and therapeutic resistance of SCLC. Increasing evidence suggests that NE plasticity in SCLC occurs through gradual transitions involving intermediate cellular states rather than abrupt lineage conversion. Recent studies further indicate that NE and non-NE populations can coexist and interconvert within the same tumor, generating substantial intratumoral heterogeneity and contributing to therapeutic resistance^36^.

In prostate cancer, therapy-induced plasticity is strongly linked to the transition from androgen receptor (AR)-dependent adenocarcinoma to AR-low, AR-null or NE prostate cancer (NEPC). This transition represents an extreme form of lineage plasticity, characterized by loss of AR signaling, acquisition of NE markers, and resistance to AR-targeted therapies. Mechanistically, lineage switching has been associated with RB1 loss and activation of neural lineage transcriptional programs, including ASCL1^37^, as well as epigenetic reprogramming mediated by factors such as EZH2^38^. EZH2 is overexpressed in advanced prostate cancer, contributes to lineage programs in both prostate adenocarcinoma and NEPC, and EZH2 inhibition has been explored as a strategy to modulate lineage state and resistance^38^. Earlier mechanistic work also showed that N-Myc drives neural lineage-associated epigenetic reprogramming and AR independence, with EZH2 inhibition partly reversing suppression of epithelial lineage genes^39^.

Melanoma represents another model of therapy-associated transcriptional plasticity. Rather than undergoing classical lineage conversion, melanoma cells frequently shift between differentiated MITF-high states and dedifferentiated or invasive AXL-high states^40,41^. These phenotypic states exhibit distinct therapeutic vulnerabilities, with dedifferentiated populations displaying increased resistance to both targeted therapy and immune checkpoint blockade^42,43^. Consistently, therapy-resistant melanomas are often associated with poorly differentiated cell states and reduced antigen presentation, linking transcriptional plasticity to immune evasion^44,45^.

Glioblastoma also exhibits marked plasticity, particularly in response to radiation and therapeutic stress. Following treatment, glioblastoma cells can transition from proneural states toward mesenchymal-like programs associated with inflammation, invasiveness, stemness and resistance. This proneural-to-mesenchymal transition is mediated by transcriptional and epigenetic reprogramming involving regulators such as STAT3, C/EBPβ and NF-κB signaling pathways. Mesenchymal transition states are strongly associated with tumor recurrence and poor prognosis, suggesting that therapy-induced plasticity contributes directly to relapse^46,47^.

Pancreatic ductal adenocarcinoma (PDAC) similarly demonstrates extensive plasticity during tumor progression and metastasis. EMT-associated programs and dedifferentiation states promote invasion, dissemination and metastatic colonization^48^. PDAC cells can adopt progenitor-like or mesenchymal phenotypes in response to environmental stress, inflammatory signaling or therapeutic pressure, thereby supporting adaptation to hostile microenvironments. These transitions are also closely linked to stem cell-like features and metastatic competence, supporting the view that plasticity is a central driver of PDAC aggressiveness^49^.

Colorectal cancer (CRC) also demonstrates profound cellular plasticity through oncofetal reprogramming and the reacquisition of CSC-like states. Several studies have shown that differentiated intestinal tumor cells can revert to stem cell-like phenotypes under regenerative or stress-associated conditions, enabling tumor propagation and therapeutic resistance. Lgr5⁻ differentiated CRC cells can regenerate Lgr5⁺ CSC populations, highlighting the dynamic and reversible nature of tumor cell hierarchy^50,51^. In addition, oncofetal reprogramming that drives fetal intestinal epithelium-like phenotypes has been identified during tumor progression and metastasis, suggesting that developmental cell-state transitions contribute to CRC aggressiveness and adaptation^52–54^.

Gastric cancer similarly exhibits marked plasticity through metaplastic reprogramming associated with tissue injury and chronic inflammation^55–57^. Persistent damage, including *Helicobacter pylori-*induced inflammation, promotes the emergence of metaplastic epithelial states that resemble regenerative or wound-healing programs^58,59^. These injury-induced transitions facilitate dedifferentiation, altered lineage commitment and the acquisition of stem cell-like properties, thereby creating a permissive environment for gastric tumor initiation and progression^60^.

Bladder cancer also exhibits cell plasticity through transitions between urothelial and divergent differentiation programs. In mixed-histology tumors, urothelial and squamous regions can arise from a common precursor, indicating that their distinct morphologies do not necessarily represent independently derived tumors. Repression of the urothelial lineage regulators (FOXA1, GATA3 and PPARG) is associated with loss of luminal identity, squamous differentiation, immune heterogeneity and reduced responsiveness to immune checkpoint blockade. These findings illustrate how disruption of lineage-defining transcriptional networks can generate clinically relevant plastic states in bladder cancer^61^.

Primary liver cancers also demonstrate marked cell plasticity between hepatocytic and biliary cell states. YAP–SOX9 signaling can promote the reprogramming of mature hepatocytes toward biliary-like states and influence whether transformed hepatocytes give rise to hepatocellular carcinoma or intrahepatic cholangiocarcinoma. More recently, PROX1 was identified as a safeguard of hepatocyte identity. Loss of PROX1 permitted hepatocellular carcinoma-to-cholangiocarcinoma lineage conversion, whereas increased PROX1 expression promoted the reverse transition^62,63^. These findings highlight the importance of lineage-fidelity regulators in determining primary liver cancer identity and heterogeneity.

### Cellular mechanisms

Cancer cell plasticity is manifested through a range of cellular processes that enable tumor cells to alter their identity and functional state. One of the most extensively studied mechanisms underlying cancer cell plasticity is EMT, a process through which epithelial cells lose their characteristic polarity and cell-cell adhesion while acquiring mesenchymal features such as enhanced motility and invasiveness^21^. Hybrid epithelial–mesenchymal states retain combinations of epithelial and mesenchymal traits and can couple migratory capacity with survival, proliferative potential and adaptability during tumor progression^22^.

In addition to EMT, phenotype switching represents another key aspect of cancer cell plasticity. Tumor cells can transition between proliferative and invasive states depending on environmental conditions, allowing them to balance growth and dissemination. This state switching is particularly evident in aggressive cancers, where distinct transcriptional programs define functionally divergent cellular states that can interconvert over time^64^.

CSC plasticity further exemplifies the reversibility of tumor cell identity. Rather than being a fixed subpopulation, CSCs can arise from more differentiated tumor cells through dedifferentiation, especially under conditions such as therapeutic stress or microenvironmental changes. Conversely, CSCs can differentiate into non-stem tumor cells, highlighting the bidirectional nature of these transitions^6,17,65^. These bidirectional transitions suggest that stemness is an adaptive cellular property regulated by both intrinsic and extrinsic factors rather than a feature restricted to a permanently defined subpopulation.

### Intratumoral heterogeneity

Cancer cell plasticity is a major driver of intratumoral heterogeneity, enabling tumor cell populations to diversify beyond what can be explained by genetic variation alone^8,17,65^. While genetic mutations contribute to clonal diversity, accumulating evidence indicates that nongenetic mechanisms allow cancer cells to adopt a wide range of phenotypic states without changes in DNA sequence^9,66^.

Plasticity generates heterogeneity by producing coexisting tumor cell populations with distinct transcriptional programs, proliferative capacities, differentiation states and sensitivities to environmental and therapeutic pressures^4,5,17,65^, thereby increasing the likelihood that a subset of tumor cells will survive under adverse conditions^9,64^.

Single-cell transcriptomic analyses have substantially expanded the understanding of intratumoral heterogeneity by resolving malignant cells into transcriptional states that are often obscured in bulk profiling, including cell-cycle-associated, differentiation-associated, EMT-like, stress-responsive and drug-tolerant programs^41,42,67–73^. Trajectory and state-transition analyses further suggest that these transcriptional programs are shaped by developmental programs, oncogenic signaling and microenvironmental stress^68,70^.

More recently, spatially resolved transcriptomic platforms have extended this framework by preserving the tissue context in which heterogeneity occurs, allowing distinct tumor cell states to be examined within native tumor architecture^74,75^. These spatial approaches further show that malignant-cell heterogeneity is not randomly distributed but can be organized across spatially structured tumor regions, linking cancer cell plasticity to local tissue organization and microenvironmental context^74,76^. Thus, the integration of single-cell and spatial transcriptomics provides a more comprehensive view of intratumoral heterogeneity by connecting cell-state variation with the tissue environments in which phenotypic diversification emerges.

### Molecular mechanisms

Cancer cell plasticity is governed by a complex network of molecular mechanisms that integrate signaling pathways, transcriptional regulators, epigenetic modifications and microenvironmental inputs^4,77^. These regulatory layers do not function independently but instead form interconnected circuits that enable reversible changes in cellular states in response to intrinsic and extrinsic cues.

Among the key drivers of plasticity are conserved developmental signaling pathways, including transforming growth factor-β (TGF-β), Wnt/β-catenin and Notch signaling^5^. These pathways play central roles in regulating EMT, stemness and cellular differentiation. TGF-β signaling is one of the most potent inducers of EMT^78^, activating transcriptional programs that suppress epithelial identity while enhancing mesenchymal traits. Similarly, Wnt/β-catenin signaling contributes to the maintenance of stem cell-like properties and supports cellular dedifferentiation^5,79,80^, whereas Notch signaling regulates cell fate decisions and promotes EMT and stemness through interactions with microenvironmental cues such as hypoxia and inflammatory signaling^22^.

At the transcriptional level, a set of master regulators orchestrates cell-state transitions^21,78,81^. Transcription factors such as SNAIL, ZEB and TWIST act as central mediators of EMT by repressing epithelial genes and activating mesenchymal gene expression programs^81^. In parallel, lineage-specific transcription factors, including SOX and KLF family members, contribute to the regulation of stemness and differentiation states^82,83^. These transcriptional networks are highly dynamic and often exhibit feedback regulation, allowing reversible transitions between cellular states.

Beyond these canonical transcriptional regulators, emerging evidence highlights the importance of cytoskeletal regulators in modulating cancer cell plasticity. Loss of CRACD, a regulator of actin dynamics, disrupts cytoskeletal organization and triggers widespread reprogramming of cellular states^35,84,85^. Recent studies demonstrate that CRACD inactivation promotes lineage plasticity by suppressing YAP–NOTCH signaling and inducing NE transcriptional programs, while simultaneously driving EZH2-dependent chromatin remodeling that represses antigen-presentation pathways and facilitates immune evasion^85^. These findings identify cytoskeletal dysregulation as an upstream regulator of cancer cell plasticity, linking changes in cellular architecture to transcriptional, epigenetic and immune programs.

Epigenetic regulation represents a central mechanism controlling cancer cell plasticity by enabling rapid and potentially reversible reconfiguration of transcriptional programs without altering the underlying DNA sequence. DNA methylation, histone acetylation and methylation, chromatin remodeling and chromatin-reader proteins collectively regulate the accessibility and activity of lineage-specific promoters and enhancers. Alterations in the expression, activity, or genomic integrity of these regulators can drive epigenetic reprogramming toward stem-like, invasive, drug-tolerant or alternative lineage states^18,24^.

Histone acetylation is a major determinant of plastic transcriptional states. The lysine acetyltransferases CBP/p300 establish active promoter and enhancer landscapes, whereas histone deacetylases remove acetyl groups and can repress lineage-defining transcriptional programs. In SCLC, CBP/p300 loss reduces histone acetylation and the expression of cell-adhesion genes, whereas HDAC inhibition can partially restore these transcriptional programs^86^. Bromodomain and extraterminal (BET) proteins, including BRD4, recognize acetylated chromatin and sustain transcription at lineage-associated and super-enhancer-regulated genes^87^.

Histone methylation and demethylation also regulate cancer cell plasticity. Loss-of-function alterations in epigenetic regulators such as *KDM6A* and *KMT2C*, together with dysregulated CBP/p300 and BRD4 activity, can reshape enhancer accessibility and weaken lineage fidelity^86–89^. KDM5A contributes to reversible drug-tolerant persister (DTP) states^18^, whereas KDM6A controls subtype switching between ASCL1- and NEUROD1-associated states in SCLC^88^. KMT2 family members, including KMT2C, maintain lineage-associated enhancer programs, and their disruption can facilitate hybrid or alternative lineage states^89^. EZH2 and LSD1 further promote dedifferentiation, NE reprogramming and therapy-adapted states through transcriptional and chromatin remodeling^38,39,90,91^.

In addition to protein-coding regulators, noncoding RNAs, including microRNAs and long noncoding RNAs, modulate plasticity-related pathways. For example, the miR-200 family forms a regulatory feedback loop with ZEB transcription factors^92,93^, controlling the balance between epithelial and mesenchymal states. Similarly, specific long noncoding RNAs influence chromatin states and transcriptional programs associated with EMT and stemness^94,95^.

### Tumor microenvironment

Cancer cell plasticity is not solely governed by intrinsic molecular programs but is also shaped by signals from the TME^4,25^. The TME comprises a complex network of cellular and noncellular components^25^, including immune cells, cancer-associated fibroblasts (CAFs), endothelial cells, ECM and soluble factors such as cytokines and growth factors. These elements provide instructive cues that regulate cell-state transitions^9^ and enable tumor cells to adapt to changing environmental conditions.

Hypoxia is a key microenvironmental factor that promotes cancer cell plasticity^96^. Oxygen deprivation stabilizes hypoxia-inducible factors (HIFs)^97^, which activate transcriptional programs associated with stemness, metabolic reprogramming and EMT^96^. Hypoxic niches within tumors enrich stem cell-like and invasive cell populations, thereby facilitating metastatic dissemination and resistance to therapy^98^.

Immune cells also play a central role in modulating plasticity^25^. Tumor-associated macrophages (TAMs) and other myeloid cells secrete cytokines such as TGF-β, interleukin-6 (IL-6) and TNF-α, which activate signaling pathways that promote EMT, stemness and immune evasion^99^. In addition to soluble factors, direct interactions between tumor and immune cells further reinforce plastic phenotypes, creating a feedback loop that sustains tumor progression^25^.

CAFs are another major component of the TME that regulates plasticity^25^. CAFs secrete ECM components, including collagen I, collagen III, fibronectin, laminin and hyaluronan, along with ECM-remodeling enzymes such as MMP2, MMP9, MMP14 and lysyl oxidase, thereby reshaping tissue architecture and increasing matrix stiffness^100–104^. Increased ECM stiffness enhances mechanotransduction signaling, including the integrin-FAK-Src and YAP/TAZ pathways, which can induce EMT and promote tumor cell invasion^101,103,105–107^. Furthermore, CAF-derived cytokines, including TGF-β and hepatocyte growth factor (HGF), influence transcriptional programs associated with stemness and cell-state transitions^108–110^.

The ECM itself is not merely a structural scaffold but an active regulator of plasticity^105^. Changes in ECM composition and organization modulate integrin signaling and cytoskeletal dynamics^101^, thereby influencing cell behavior and fate decisions. Mechanical cues such as stiffness and tension have been shown to drive phenotypic switching and enhance metastatic potential^106^.

Endothelial cells and vascular niches also contribute to plasticity by providing angiocrine signals (that is, endothelial cell-derived paracrine factors) that maintain stem cell-like states and promote tumor cell survival^111,112^. Endothelial cells provide signals such as Notch ligands and IL-6 that activate pathways associated with self-renewal, EMT and cell-state transitions^112–115^. Endothelial-derived Notch signaling and IL-6/STAT3 activation have been implicated in maintaining CSC phenotypes and therapy resistance. Hypoxic vascular niches further enhance plasticity through HIF-dependent transcriptional reprogramming, promoting EMT, metabolic adaptation and survival under stress conditions^115^. Thus, the TME serves as a dynamic regulatory network that integrates biochemical and mechanical signals to sustain cancer cell plasticity, thereby facilitating tumor adaptation, metastasis and therapy resistance^116,117^.

### Cell plasticity in tumor initiation

Cell plasticity is increasingly recognized as an important driver of tumor initiation, rather than merely a consequence of tumor progression^4^. Under physiological conditions, adult epithelial tissues are maintained by relatively stable lineage hierarchies. However, tissue injury, chronic inflammation and environmental stress can destabilize these lineage constraints and induce differentiated or lineage-committed cells to reacquire progenitor-like features^26,118^. This process may occur through dedifferentiation, transdifferentiation or metaplastic reprogramming, thereby generating cellular states that are more permissive to oncogenic transformation^3–5^.

In the intestinal epithelium, tumor initiation has been linked to the transformation of stem cells, particularly Lgr5^+^ crypt base columnar cells^119,120^. However, accumulating evidence indicates that non-stem or differentiated epithelial cells can also acquire tumor-initiating capacity under specific inflammatory or regenerative contexts^121–123^. For example, activation of NF-κB signaling can cooperate with Wnt pathway activation to induce dedifferentiation of intestinal epithelial non-stem cells into tumor-initiating cells^26,124^. This suggests that inflammation can expand the pool of cells capable of initiating tumors by lowering lineage barriers and promoting stem cell-like reprogramming^26,124,125^.

Recent lineage-tracing and genetic barcoding approaches have strengthened this view by directly linking precursor cell identity, clonal expansion and plastic state transitions during tumor evolution. When combined with single-cell transcriptomics, these approaches enable cell lineage trajectories to be reconstructed alongside transcriptional states, revealing clonal trajectories and the diversification of initially defined precursor populations over time^126–128^.

Regeneration-associated plasticity also contributes to early tumorigenesis. During colonic injury, epithelial cells can undergo YAP/TAZ-dependent reprogramming toward a fetal-like regenerative state^52,129^. This transient state is required for tissue repair. However, in the presence of oncogenic alterations, such as *APC* loss or *KRAS* activation, regenerative programs may become aberrantly stabilized, thereby supporting neoplastic transformation^26,27^. Thus, tumor initiation can arise from the inappropriate persistence of wound-healing or fetal-like programs rather than from genetic alterations alone.

Similar principles have been observed in other epithelial tissues^4,5,130^. In the pancreas, chronic inflammation promotes acinar-to-ductal metaplasia, a plasticity-associated reprogramming event in which differentiated acinar cells acquire duct-like features^131,132^. Although this process can be reversible during normal repair, persistent inflammation combined with oncogenic *KRAS* activation promotes progression from metaplasia to pancreatic intraepithelial neoplasia and PDAC^133,134^. Macrophage-derived inflammatory cytokines have also been shown to drive acinar-to-ductal metaplasia, highlighting the role of the microenvironment in shaping tumor-initiating plastic states^135^.

Oncogenic alterations can further enhance cellular plasticity by disrupting lineage fidelity. In the mammary epithelium, oncogenic *PIK3CA* (H1047R) mutations have been shown to induce multipotency in lineage-restricted mammary cells, generating heterogeneous tumor phenotypes from otherwise committed cell populations^136,137^. Similarly, dysregulation of tumor suppressors such as *TP53*, *RB1* and *PTEN* can interfere with normal differentiation programs and facilitate lineage switching or the acquisition of progenitor-like states^138–141^. These findings indicate that tumor initiation is not determined solely by the cell of origin but is also shaped by the plastic potential of the transformed cell and its microenvironmental context, which together influence tumor phenotype and heterogeneity.

### Cell plasticity in metastasis

**Fig. 2:**
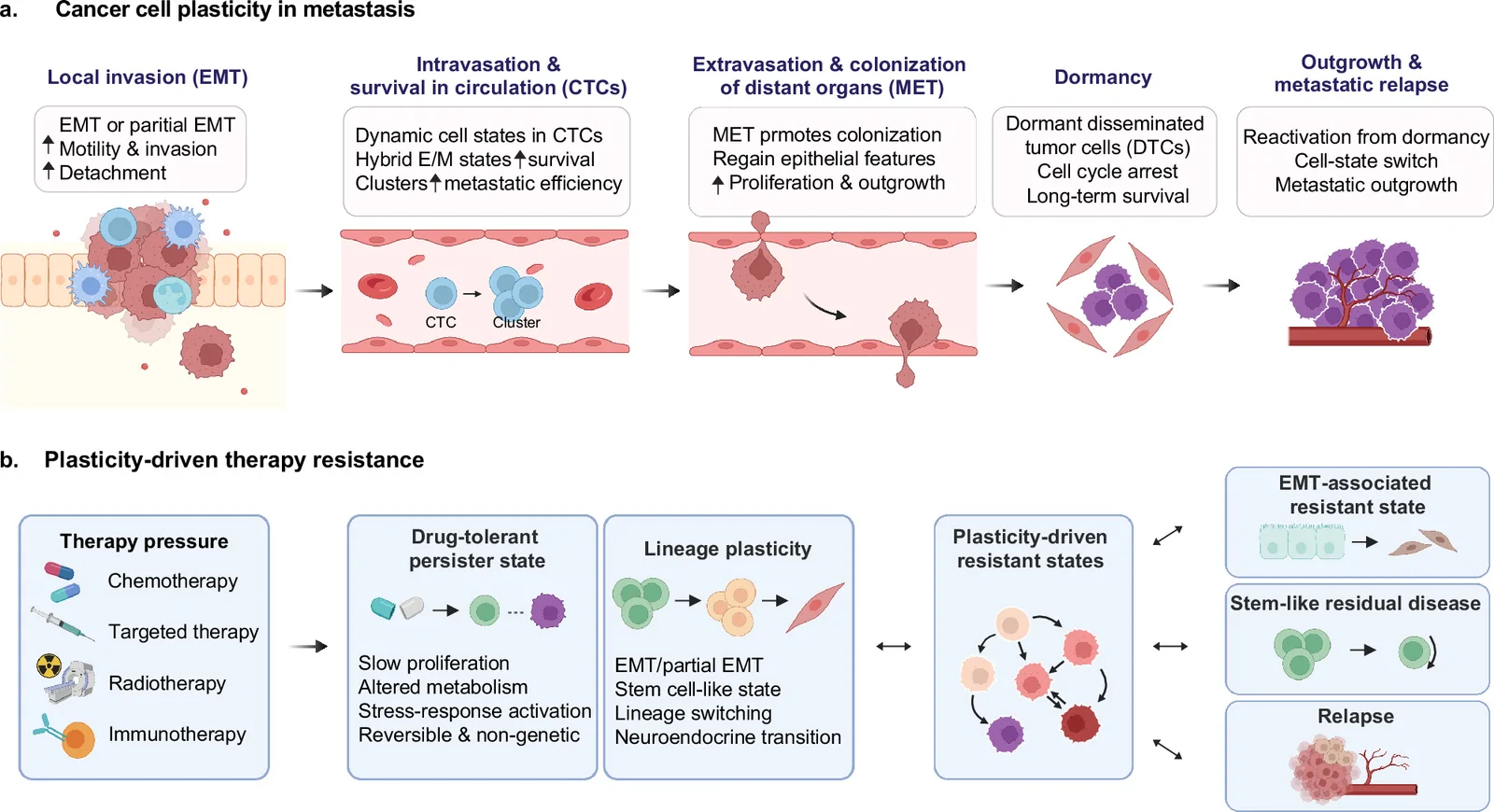
Cancer cell plasticity in metastasis and therapy resistance. **a**, Cancer cell plasticity supports multiple stages of the metastatic cascade through dynamic and reversible cell-state transitions. During local invasion and dissemination, epithelial tumor cells acquire partial or mesenchymal traits that enhance motility and invasiveness. Hybrid epithelial–mesenchymal states can promote survival in circulation, whereas reversion toward an epithelial state through MET facilitates colonization and proliferative outgrowth at distant organs. Disseminated tumor cells may also enter dormant states and subsequently reactivate in response to microenvironmental signals, contributing to metastatic relapse. **b**, Therapeutic pressure imposed by chemotherapy, targeted therapy, and immunotherapy can induce adaptive and reversible cancer cell-state transitions. Under treatment stress, tumor cells may enter DTP states or undergo lineage plasticity, generating heterogeneous resistant populations. These plasticity-driven resistant states are associated with EMT-related programs, stem cell-like residual disease and the survival of therapy-adapted cells, ultimately contributing to tumor recurrence and relapse.

Cancer metastasis is a multistep process requiring tumor cells to adapt to distinct microenvironmental challenges at each stage^17^. Rather than being driven by a fixed population of metastatic cells, accumulating evidence indicates that metastasis is enabled by continuous and reversible cell-state transitions governed by cancer cell plasticity^4,9^ (Fig. 2a).

During local invasion, epithelial tumor cells undergo phenotypic changes that enhance motility and invasiveness. This process is frequently associated with EMT, which allows tumor cells to detach from the primary tumor mass and invade surrounding tissues^21,78^. Importantly, partial or hybrid EMT states have been shown to confer greater metastatic potential than fully mesenchymal states, as they retain a balance between migratory capacity and proliferative potential^20–22^.

Following invasion, tumor cells intravasate into the circulation as circulating tumor cells (CTCs), where they encounter substantial stress, including shear forces and oxidative damage^17^. At this stage, plasticity enables tumor cells to adopt stress-resistant phenotypes. CTCs often undergo dynamic transitions between epithelial and mesenchymal traits, and cells in hybrid states exhibit enhanced survival and increased metastatic competence^20,22^. In addition, clustered CTCs exhibit greater metastatic efficiency^142^, further highlighting the importance of collective plasticity^23^.

Upon reaching distant organs, tumor cells must extravasate and adapt to a new microenvironment. At this stage, a reversal of EMT, known as mesenchymal–epithelial transition (MET), is often required for effective colonization and outgrowth^17^. This transition allows tumor cells to regain proliferative capacity and establish secondary tumors. The ability to switch between EMT and MET states is therefore critical for successful metastasis^4^.

A subset of disseminated tumor cells can enter a dormant state, characterized by cell-cycle arrest and long-term survival without active proliferation^4,17^. Plasticity enables these cells to enter and exit dormancy in response to microenvironmental cues. Dormant cells are often resistant to therapy and can later reactivate, leading to metastatic relapse even after prolonged periods of clinical remission.

### Plasticity-driven therapy resistance

Cancer cell plasticity has emerged as a major contributor to therapeutic resistance, allowing tumor cells to survive under treatment pressure without requiring permanent genetic alterations^9^. Unlike classical resistance mechanisms driven by the selection of pre-existing genetic mutations, plasticity enables tumor cells to adopt alternative cellular states that are less sensitive to therapy^23^ (Fig. 2b).

One of the key manifestations of plasticity-mediated resistance is the emergence of DTP cells. These cells do not harbor stable resistance-conferring mutations but instead adopt transient phenotypic states that support survival during treatment^9,18^. DTP states are often characterized by reduced proliferation, altered metabolic activity and activation of stress-response pathways. Importantly, these cells can reenter the cell cycle upon drug withdrawal, giving rise to tumor regrowth and relapse^9,18^.

Epigenetic reprogramming plays a central role in the establishment and maintenance of drug-tolerant states. Changes in chromatin accessibility and histone modifications enable shifts in transcriptional programs without altering the underlying DNA sequence^4^. This reversibility allows tumor cells to withstand therapeutic stress and later return to proliferative states, highlighting the importance of nongenetic mechanisms in resistance^9^.

Plasticity-driven resistance is also closely linked to specific cell-state transitions. EMT-associated states have been shown to confer resistance to a wide range of therapies, including chemotherapy, targeted therapy and immunotherapy^23^. Similarly, stem cell-like tumor cells exhibit enhanced survival capacity and are often less sensitive to treatment, contributing to minimal residual disease^4^.

In addition to intrinsic mechanisms, interactions with the TME further reinforce therapy resistance. Stromal cells, immune components and hypoxic conditions provide protective niches that support the survival of DTP cells. These extrinsic signals can induce or stabilize resistant phenotypes, creating a feedback loop that sustains tumor persistence^25,116,117^.

### Therapeutic targeting of cancer cell plasticity

The dynamic and reversible nature of cancer cell plasticity presents both therapeutic opportunities and challenges. Emerging strategies aim to prevent maladaptive state transitions, restore lineage differentiation or selectively eliminate plastic and therapy-tolerant states. Epigenetic regulators are particularly attractive targets because they coordinate the transcriptional programs underlying lineage switching. In treatment-resistant prostate cancer models, targeting EZH2 can reverse lineage-infidelity programs and restore sensitivity to AR-directed therapy^143^. LSD1 inhibition can similarly disrupt plastic states by activating NOTCH signaling and suppressing ASCL1-dependent NE plasticity in SCLC, while allosteric LSD1 inhibition can restore TP53 signaling and reduce NEPC growth^90,91^. BET protein inhibition has also been shown to suppress treatment-emergent NE lineage programs in prostate cancer^144^. In addition to epigenetic therapies, targeting plasticity-inducing signals from the TME or exploiting state-specific dependencies may prevent the establishment or persistence of resistant populations. Because these regulators may have context-dependent effects on cell identity, biomarker-guided treatment combinations and appropriate therapeutic timing will be important for translating plasticity-directed strategies into clinical benefit.

### Technological advances in the study of cell plasticity

**Fig. 3:**
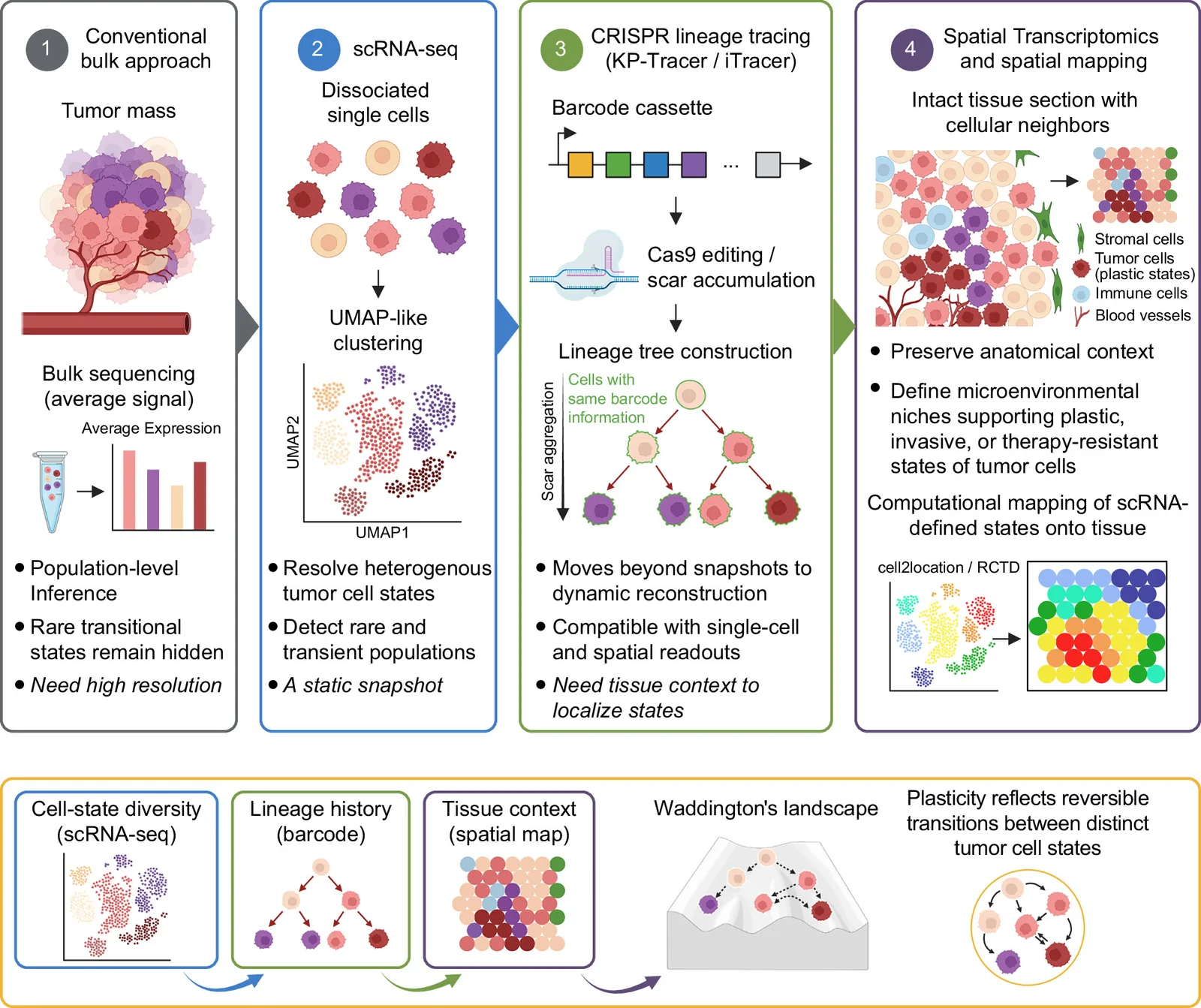
Advances in technology for studying cell plasticity. (1) Conventional bulk profiling provides an average molecular signal from mixed malignant and non-malignant populations within the tumor mass. Although this approach enables population-level inference of cancer-associated pathways, it can obscure rare transitional states, minor plastic subpopulations and cell-type-specific programs. (2) scRNA-seq improves this resolution by profiling dissociated individual cells and separating heterogeneous tumor cell states through clustering and dimensionality reduction. This enables the detection of rare, transient, proliferative, invasive, stem cell-like or drug-tolerant populations that are often missed by bulk data. However, because scRNA-seq captures a static snapshot, it cannot by itself determine whether distinct transcriptional states represent interconverting plastic states, clonally related diversification, metastatic outgrowth or unrelated tumor subpopulations. (3) To overcome this snapshot limitation, lineage-recording technologies add clonal history to single-cell cell-state profiles. In KP-Tracer/iTracer-based systems, Cas9-mediated barcode editing generates heritable scar patterns that accumulate during tumor cell divisions. Cells with related barcode scars can be grouped to infer clonal relationships and reconstruct lineage trees. By linking lineage history with transcriptomic states, these approaches help distinguish plastic transitions, clonal diversification, metastatic dissemination and unrelated tumor heterogeneity, while also providing barcode-defined populations that can be integrated with spatial transcriptomic readouts. (4) This lineage-resolved view can be further extended by spatial transcriptomics and spatial mapping, which place tumor cell states back into their native tissue architecture. By preserving the spatial organization of tumor, stromal, vascular and immune populations, these methods identify microenvironmental niches associated with plastic, invasive or therapy-resistant tumor states. Computational mapping tools such as cell2location and RCTD can project scRNA-seq-defined cell types or cell states onto spatial data, linking transcriptional plasticity to local tissue structure and neighboring cell populations. Single-cell state diversity, barcode-derived lineage history and spatial tissue context provide complementary layers for interpreting cancer cell plasticity, with each layer resolving a different aspect of the process: transcriptional state variation, clonal ancestry and tissue localization within the TME. When integrated, these modalities help to distinguish whether heterogeneous tumor cell states represent transient plastic programs, clonally related state diversification or spatially restricted phenotypes shaped by local tissue context. These layers can be summarized using Waddington’s landscape model, in which tumor cells are visualized as occupying distinct transcriptional states and moving between alternative states through reversible plastic transitions. In this representation, the landscape serves as a graphical depiction of cell-state plasticity, while lineage tracing and spatial transcriptomics provide additional information about the clonal history and anatomical context associated with those states.

Recent advances in single-cell, lineage-tracing and spatial transcriptomic technologies have transformed the study of cancer cell plasticity from population-level inference to direct measurement of dynamic cell-state variation (Fig. 3). Earlier studies largely inferred plasticity from bulk transcriptomic changes or phenotypic assays, which limited the ability to distinguish rare transitional states from dominant tumor populations. The advent of single-cell RNA sequencing (scRNA-seq) addressed this limitation by resolving heterogeneous tumor cell states at single-cell resolution. These studies have shown that cancer cells often occupy a continuum of transcriptional states rather than discrete and fixed identities, revealing rare, transient and intermediate populations that are frequently missed by conventional bulk analyses^41,42,67–73^.

However, scRNA-seq alone provides a snapshot of tumor heterogeneity and cannot fully determine whether transcriptionally distinct states represent interconverting populations, independent subclones or separate differentiation trajectories. This limitation has motivated the integration of lineage-tracing technologies with single-cell profiling. Evolving barcode systems such as KP-Tracer enable reconstruction of cell lineage relationships from progressively accumulated genetic marks while simultaneously recovering single-cell transcriptional profiles. In this design, clonal history and cell-state identity are measured in the same tumor cells, allowing tumor evolution, metastatic dissemination and plastic state transitions to be analyzed within a unified framework^128^. Complementary inducible recording systems such as iTracer extend this concept by combining reporter barcodes with CRISPR–Cas9-mediated genetic scarring, thereby enabling temporal control of lineage recording and compatibility with both single-cell and spatial transcriptomic readouts. This dual-channel strategy is particularly useful for linking cellular ancestry to transcriptional identity and tissue organization^126,127^. Together, these lineage-resolved approaches provide a methodological bridge between static single-cell snapshots and the temporal reconstruction of cancer cell plasticity.

Although lineage-resolved scRNA-seq provides temporal and clonal information, dissociation-based methods remove tumor cells from their native anatomical context. This limitation is important because cancer cell plasticity is also shaped by extrinsic cues, including hypoxia, stromal signaling, immune pressure, ECM organization and vascular niches. Spatial transcriptomic technologies address this gap by preserving the tissue architecture in which plastic states arise. These approaches have revealed regional variation in tumor cell states and begun to define how specific tumor niches support invasive, stem cell-like, plastic or therapy-resistant phenotypes^12,74–76,145,146^. In parallel, computational integration methods now allow scRNA-seq-defined cell states to be mapped onto spatial transcriptomic data. Approaches such as cell2location and robust cell type decomposition (RCTD) use single-cell reference profiles to infer the spatial distribution of fine-grained cell types or states within intact tissues, thereby linking molecular plasticity to local tissue organization and neighboring cell populations^147,148^.

Together, these technologies provide complementary dimensions for studying cancer cell plasticity: scRNA-seq defines the diversity of cellular states, lineage tracing reconstructs their ancestry and transition history, and spatial transcriptomics places them within the microenvironmental niches that regulate their behavior. Models inspired by the Waddington landscape help integrate these dimensions by viewing tumor cells as moving across a transcriptional or epigenetic landscape composed of distinct cell differentiation states^14–16^. In this context, plasticity can be interpreted not simply as loss of differentiation but as regulated movement between alternative cell states with different degrees of stability, lineage constraint and environmental dependence^14–16^. This integrated perspective allows cancer cell plasticity to be studied as a lineage-informed and spatially organized process rather than a static transcriptional phenotype.

## Concluding remarks

Cancer cell plasticity reflects the capacity of tumor cells to adapt to intrinsic and extrinsic pressures through reversible changes in cellular identity. By supporting transitions among stem cell-like, epithelial, mesenchymal, dormant and drug-tolerant states, plasticity provides a mechanistic link between intratumoral heterogeneity, metastatic dissemination and therapeutic resistance. This perspective extends genetic and clonal evolution frameworks by emphasizing the contribution of nongenetic, epigenetic and microenvironmentally regulated state transitions to cancer progression. Recent advances in single-cell and spatial transcriptomics distinguish reversible cell-state transitions from clonal selection and identify the niches and regulatory programs that stabilize or destabilize plastic states. Beyond malignant cells, plasticity also occurs in stromal and immune cell populations within the tumor niche, including CAFs, T cells and macrophages, highlighting the need for further investigation. Moving forward, targeted pharmacological strategies that exploit cancer cell plasticity hold immense promise to block maladaptive cell-state transitions, eliminate drug-tolerant or dormant populations, and disrupt microenvironmental signals sustaining tumor adaptability. Such approaches may offer a compelling path toward preventing metastasis, overcoming therapy resistance and achieving more durable clinical responses.
